# Disseminated *Nocardia farcinica* involves the spinal cord: a case report and review of the literature

**DOI:** 10.1186/s12879-021-06905-y

**Published:** 2021-12-07

**Authors:** Jing Wu, Xiaoran Li, Tao Zhang, Xin Lin, Yu-Chen Chen

**Affiliations:** 1 Department of Radiology, Nanjing First Hospital, Nanjing Medical University, No.68, Changle Road, Nanjing, 21006 People’s Republic of China; 2 Department of Radiology, Nanjing Gaochun People’s Hospital, Nanjing, Jiangsu People’s Republic of China; 3 Department of Laboratory, Nanjing First Hospital, Nanjing Medical University, Nanjing, Jiangsu People’s Republic of China

**Keywords:** *Nocardia farcinica*, Spinal cord, Case

## Abstract

**Background:**

*Nocardia* is a relatively rare opportunistic pathogenic bacteria group, commonly seen in patients with immunocompromised or defective immune system. It can affect multiple organs of the body and cause disseminated infection, among which most occurs in the lung, secondly in the nervous system, soft tissues, rare in the spinal cord and pituitary. No case has been reported involving lung, spinal cord, skin and pituitary gland at the same time.

**Case presentation:**

We report a 55-year-old female with *Nocardia* infection involving the lung, skin, spinal cord, and pituitary gland. The patient underwent a full set of imaging examinations and showed typical imaging findings. Chest computed tomography (CT) showed multiple nodules with cavities in the lungs. The magnetic resonance imaging (MRI) of the vertebral body showed abnormal signal of the entire spinal cord with cavity formation and ring enhancement. The subcutaneous nodules of the abdomen were punctured under ultrasound. Through the etiological tissue culture of subcutaneous nodules and the second generation sequencing of cerebrospinal fluid, the diagnosis was finally confirmed.

**Conclusion:**

Disseminated *Nocardiosis* is an uncommon disease. This article will report a rare case of disseminated *Nocardiosis* simultaneously involving the lung, spinal cord, subcutaneous soft tissue and pituitary gland, especially with neuropathy as the initial symptom. Imaging is helpful for the early diagnosis of the disease and pathological and microbiological examinations are helpful for its confirming.

## Background

*Nocardia* is a weakly acidic, Gram-positive aerobic actinomycete that is widely present in soil, water, air and saprophytes [1]. Nocard first described it in 1888 [2]. In 1890, Eppinger first reported human infection with *Nocardiasis* [3]. So far, there are about 80 species of *Nocardia*, and about 33% of them can infect humans [4]. The common species are *Nocardia asteroides*, *Nocardia brasiliensis* and so on. *Nocardia asteroides* are the most common, accounting for about 70% [5]. *Nocardia farcinica*, accounting for 24.5% of all *Nocardia* infections [6], is more likely to cause disseminated infection and higher mortality [3]. Because it is difficult to be detected in time, the diagnosis is more difficult, which can lead to delays in treatment and poor prognosis of patients. This case introduces *Nocardia* infection involving the lung, skin, spinal cord, and pituitary gland. Its typical imaging findings will help early diagnosis.

## Case presentation

A 55-year-old woman was admitted to our hospital on 27 July 2021 due to severe chest and back pain and weakness of lower extremities for 11 days. She also complained of urinary and fecal incontinence. Fever was not reported since the onset. She has had a history of hypertension, diabetes and depression. In addition, she used glucocorticoids and immunosuppressant for 2 months because of immune thrombocytopenic purpura. Physical examination revealed symmetrically completely paralysis in the lower extremities, decreased muscle tension, absence of knee and ankle reflexes, and the presence of bilateral extensor plantar reflex. Several subcutaneous lumps in the lower abdomen on the left side were also revealed. The patient’s blood routine, electrolytes, liver and kidney functions were normal, and the test for syphilis and human immunodeficiency virus (HIV) was negative. Albumin is reduced to 26.7 g/L (40–55 g/L as the normal value). Consecutive cerebrospinal fluid (CSF) examinations showed a normal range in white blood cell (WBC) count and significant increase in protein concentration.

**Fig. 1:**
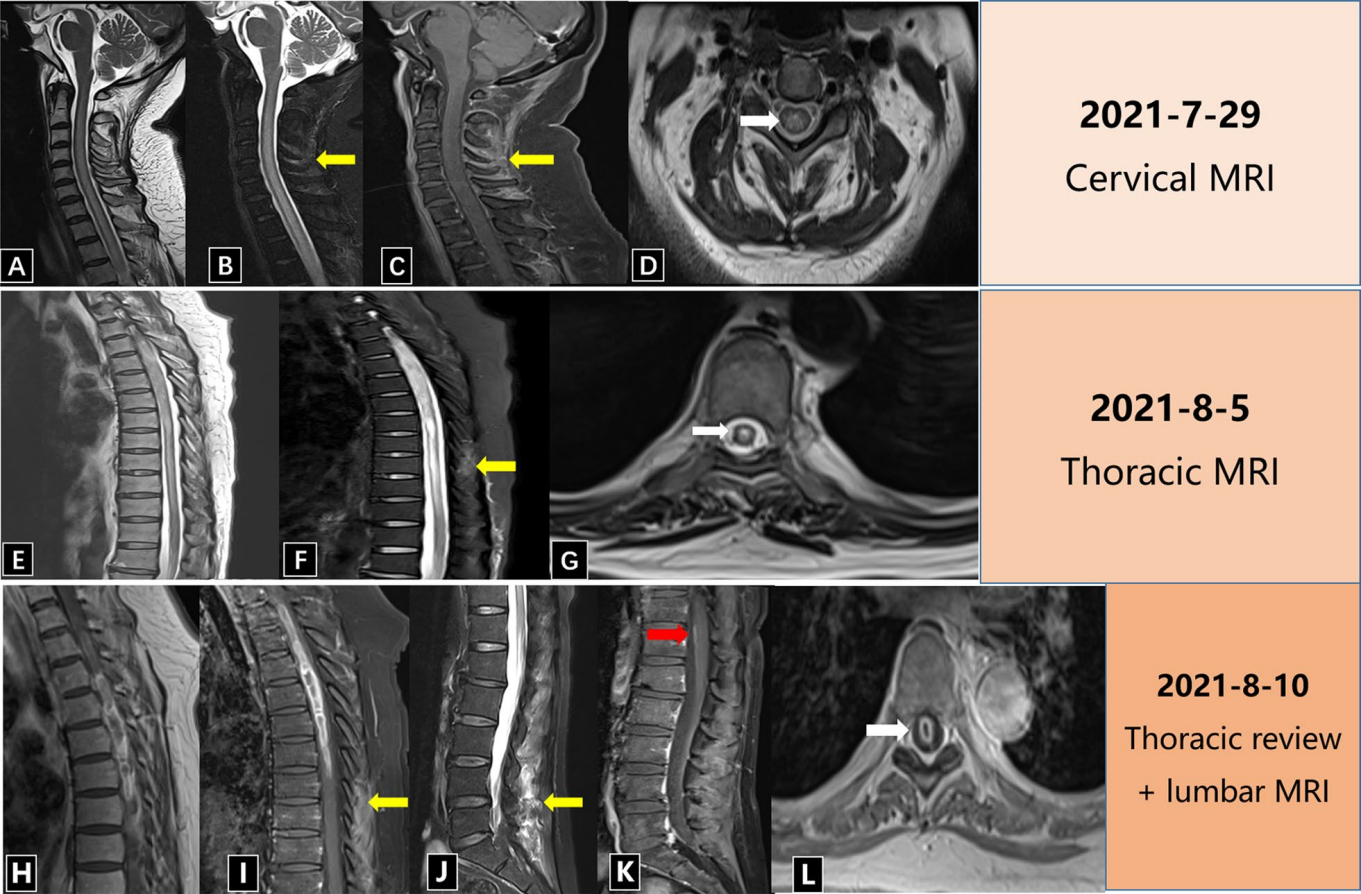
The patient’s cervical, thoracic and lumbar spine MRI. **A**–**D** are the cervical spine MRI on July 29, 2021. **A** is T2WI, and **B** is the T2 fat suppression sequence. It shows hyperintensity in the entire cervical spinal cord. The posterior soft tissue shows patchy hyperintensity as yellow arrow. **C** (T1WI enhanced sequence) shows the posterior soft tissue obviously enhanced in patches as yellow arrow. **D** (axial position of the cervical spine of T2WI) shows high signal in the spinal cord as white arrow. **E**–**G** is the thoracic MRI on August 5, 2021. **E** is T2WI, **F** is the T2 fat suppression sequence, showing the entire thoracic spinal cord with high signal intensity, and the yellow arrow shows the posterior soft tissue with patchy high signal. **G** shows the axial T2WI of the thoracic spine with high signal in the thoracic spinal cord as white arrow. **H**–**L** is the thoracic follow-up MRI and lumbar spine MRI on August 10, 2021. **H** (Thoracic spine of coronal T2WI) compared with the previous image (**E**), there is a cavity in the center of the thoracic spinal. **I** (T1WI enhancement), the intrathoracic spinal cord lesion shows a long strip-shaped edge enhancement, and the posterior soft tissue shows patch-like enhancement. **J** (T2WI fat suppression sequence of the lumbar spine) shows the posterior soft tissue with patchy hyperintensity. **K** (coronal enhanced sequence) shows the streak-shaped spinal membrane enhancement as red arrow. **L** is the axial position, showing the ring-shaped enhancement high signal in the thoracic spinal cord as white arrow

**Fig. 2:**
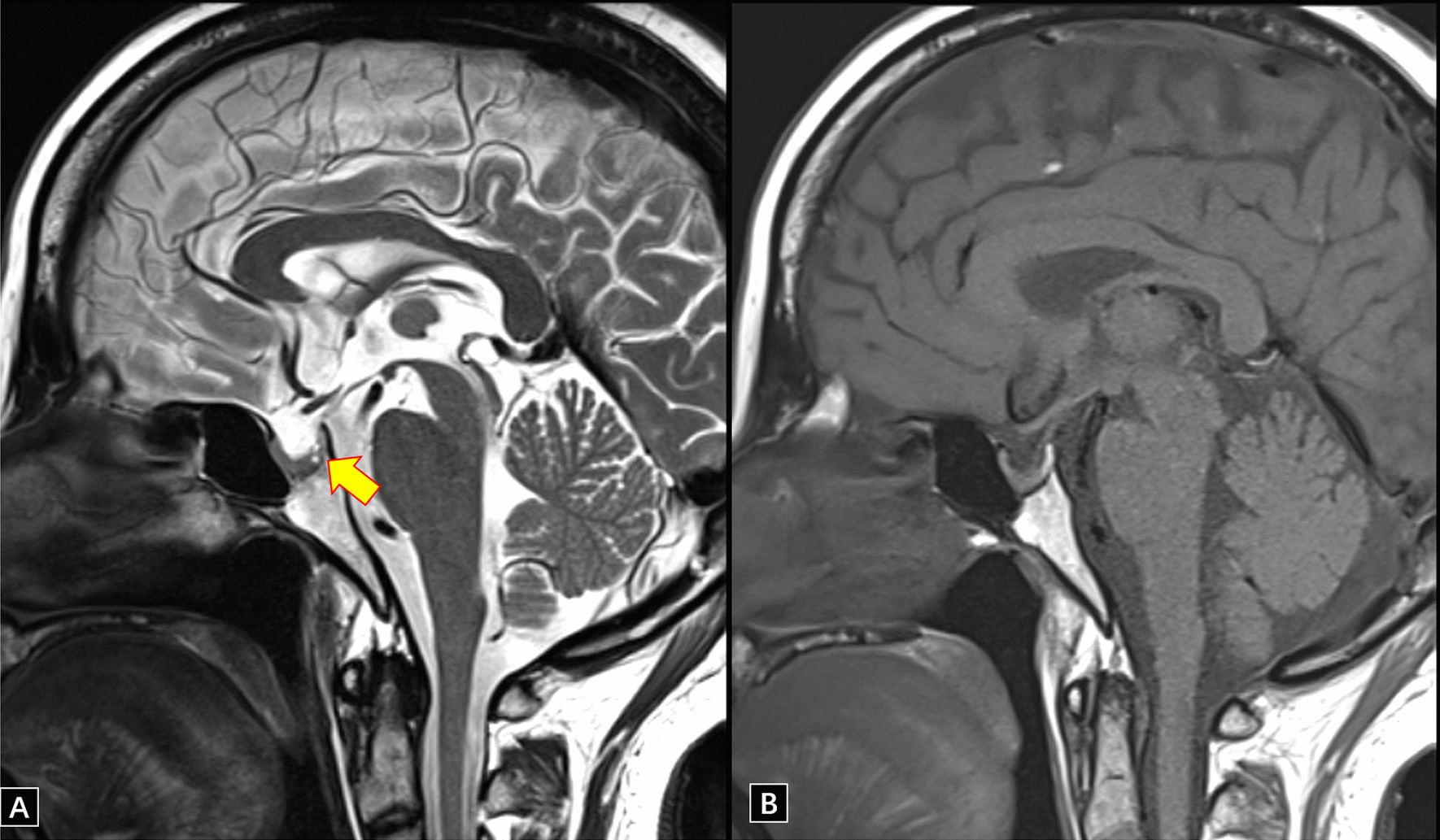
The patient’s pituitary MRI on August 2, 2021. **A** is a T2WI sagittal image, showing two nodular T2 hyperintensity shadows on the back of the pituitary gland as indicated by the arrow, and **B** is T1WI with no obvious lesions

The cranial and cervical spine, thoracic spine and lumbar spine magnetic resonance imaging (MRI) showed abnormal signal in the spinal cord and the pituitary. MRI examinations were performed for three times from July 29, 2021 to August 10, 2021 in Figs. 1 and 2. The result shows abnormal signals in the entire spinal cord (cervical spinal cord and thoracic spinal cord) in Fig. 1A–G. Follow-up MRI of the thoracic spine (Fig. 1E VS Fig. 1H) shows cavitation changes in the thoracic spinal cord, suggesting that the lesion has changed in a short period of time, and necrosis and cavitation in the center suggesting infectious lesions. In addition, the posterior soft tissue of the cervical, thoracic and lumbar spine all showed patchy abnormal signals with enhancement, suggesting inflammatory changes. Meanwhile, two nodules were seen on the back of the pituitary gland with T2 hyperintensity in Fig. 2.

**Fig. 3:**
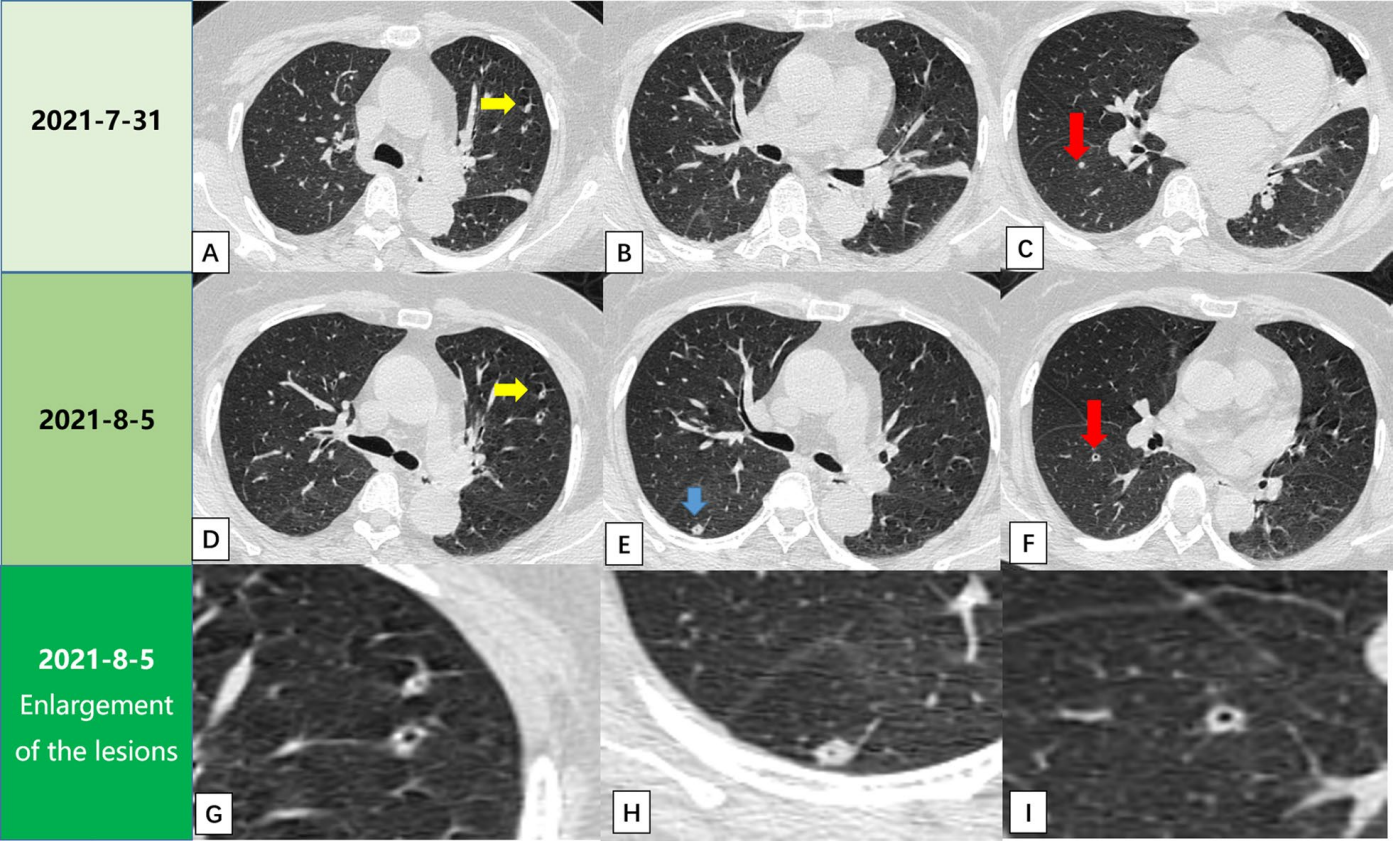
The comparison between the two CT examinations of the patient. **A**–**C** are the initial chest CT on July 31, 2021, showing patchy high-density shadows in the lingual segment of the left upper lobe, and multiple nodules in both lungs, as shown by the yellow arrow in **A** and the red arrow in **C**. **D**–**F** is a follow-up CT with the same slice as the previous images. Compared with **A**, **D** shows small cavity in the left pulmonary nodule, as shown in the enlarged **G**. Compared with **B**, **E** shows a new nodule in the right pulmonary lung as the blue arrow. The enlargement image in **H** shows a small cavity in the center. Compared with **C**, **F** shows the patchy high density has disappeared in the left lung and a small cavity appears in the nodule of the right lower lobe. The enlargement image in **I** shows a small cavity in the center

The patient underwent chest computed tomography (CT) examinations as shown in Fig. 3. Figure 3A–C is the initial chest CT on July 31, 2021, showing a patchy high-density shadow in the left lung and multiple nodules in bilateral pulmonary. Figure 3D–F is follow-up CT on August 5, 2021, which shows the patchy high-density shadow disappears of the left lung, and cavities appear in nodules suggesting that the lesions have changed in a short time, and nodules with cavity suggesting the infection.

**Fig. 4:**
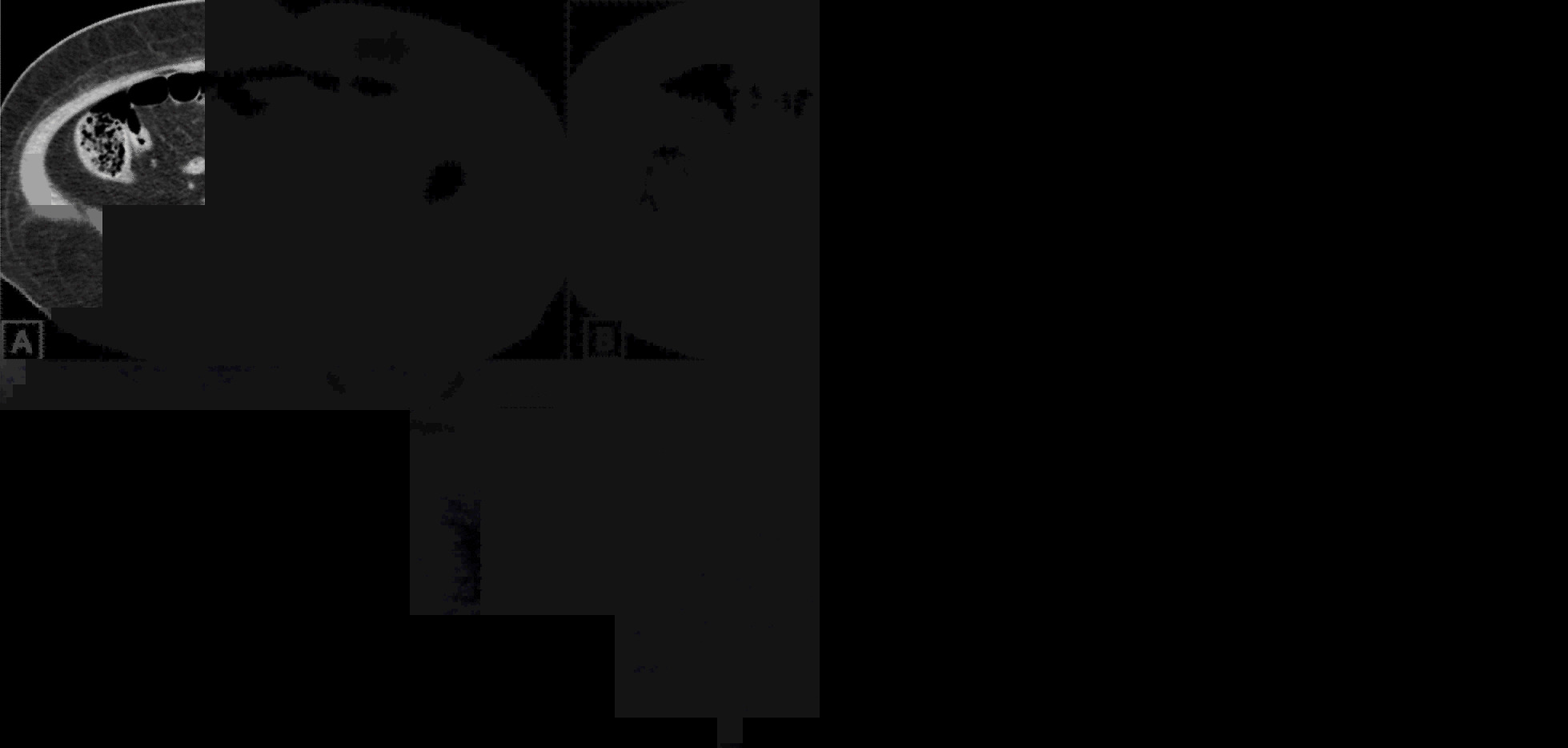
CT images of the patient's abdomen, B-ultrasound and puncture pathology of the subcutaneous nodules of the abdominal wall. **A**, **B** show two nodules under the skin of the abdominal wall, as shown by the yellow and red arrows. The blue arrow shows the subcutaneous strip of high-density shadows, suggesting inflammation. **C** Shows enlarged spleen. **D** is a B-ultrasonic image of abdominal wall nodules. There is a 37 mm × 20 mm, 24 mm × 14 mm mixed echo group in the fat layer of the left abdominal wall, with irregular shape, uneven internal echo, and insignificant internal blood flow signal. Under ultrasound-guided puncture of the left abdominal wall nodule, the pathological image is shown in **E** (Hematoxylin and eosin staining of the nodule, original magnification × 200. Scale bar = 50 μm). Microscopic images were taken with VS200 Slide Scanner (Olympus, Tokyo, Japan) at a resolution of 1880 × 1048 pixels, analyzed with Olyvia software (Olympus). A large number of inflammatory cells including lymphocytes and neutrophils are seen, as well as some pus cells which are fragments of inflammatory cells. A few fibroblasts can be seen among inflammatory cells. Colony smear showed filamentous fragment forms of Acid fast bacilli by Acid-fast stain in **F** (Original magnification × 1000. Scale bar = 5.0 μm). Microscopic images were taken with VS200 Slide Scanner (Olympus, Tokyo, Japan) at a resolution of 1560 × 920 pixels, analyzed with Olyvia software (Olympus)

**Fig. 5:**
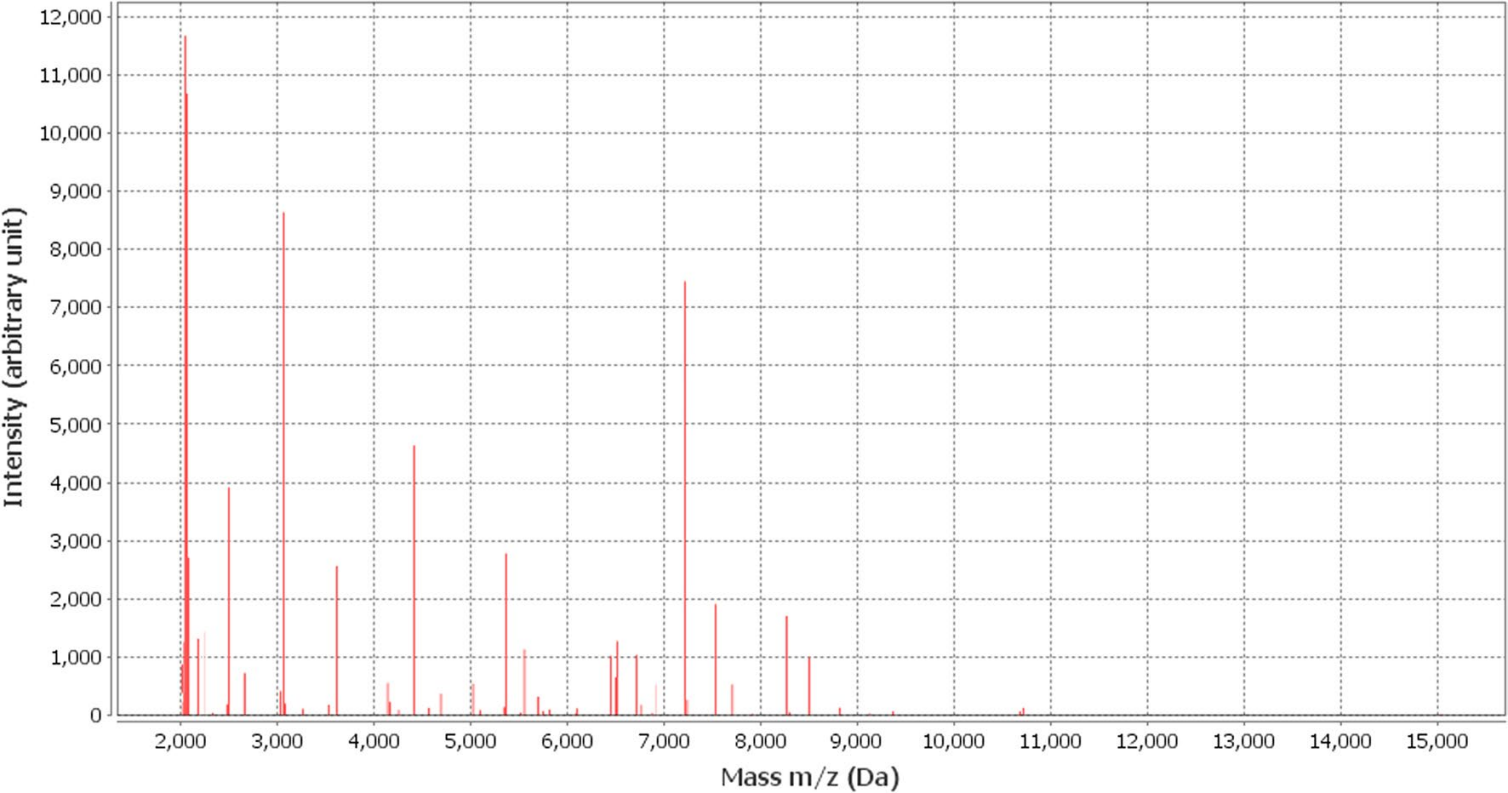
The bacterial protein spectra obtained were analyzed by the matrix-assisted laser desorption/ionization time of flight mass spectrometry. The unique *Nocardia farcinica* was identified using mass spectrometry

On July 31, 2021, the patient underwent abdominal CT examination and found two nodules under the skin of the left lower abdominal wall, as shown in Fig. 4A, B. There are also strips of high density around, suggesting inflammatory changes. Figure 4C shows the spleen become large. B-ultrasound examination of abdominal wall nodules in Fig. 4D shows mixed echo bolus. After ultrasound puncture, the pathology in Fig. 4E shows a large number of inflammatory cells suggesting infection (HE × 400; Olympus, Tokyo, Japan). The acid-fast stain showed partially acid-fast bacilli in Fig. 4F (× 1000). Culture of the pus and blood fluid revealed an uncommon microorganism, termed *Nocardia farcinica*. *Nocardia farcinica* protein spectra obtained was analyzed by mass spectrometry in Fig. 5. The results confirmed that it is *Nocardia* with the coincidence rate 99.9%. No positive discovery was in the culture of CSF. However, second generation sequencing of the CSF revealed the same microorganism.

## Discussion and conclusions

*Nocardia* infections often occur in patients with weak or defective immune function. Risk factors include acquired immunodeficiency syndrome (AIDS), organ transplantation, tumor radiotherapy and chemotherapy, or long-term use of immunosuppressive agents [7, 8]. It is less common in people with normal immune function [9]. This patient used glucocorticoids and immunosuppressant for 2 months, which may cause her infection. *Nocardia* usually enters the human body through the respiratory tract, digestive tract or broken skin, which can cause localized infection. It can also spreads to multiple organs throughout the body via the blood circulation, most likely to occur in the lung [10], common clinical symptoms are cough, sputum, fever, fatigue, etc. [11]. However, this patient was admitted to the hospital for neurological symptoms rather than respiratory symptoms, which is relatively rare. *Nocardia farcinica* invade the central nervous system, clinically often manifested as fever and headache caused by brain abscess, occasionally seen in the spinal cord and meninges [12, 13].Therefore, this is a rare case that deserves attention.

The common CT manifestations of *Nocardia* pneumonia are pulmonary nodules, diffuse or localized lung infiltration, lung abscess, and pleural effusion [14, 15]. The predominant image is granuloma or nodule with cavity formation. Our case is consistent with this pattern, that is multiple cavities occurring and changing over a short period of time. The next is the nervous system, common imaging findings include brain abscess, granulomatous formation or diffuse infiltration [16, 17], meningitis, epidural and paraspinal abscesses [18, 19]. This case has no typical brain MRI performance. However, lesions in the spinal cord, and the cavity formation and annular enhancement appeared during the check-up MRI of the thoracic vertebra, which proved that it was a suppurative bacterial infection. Meanwhile, enhancement of the spinal membrane and the abnormal signal in the soft tissue of the back also reflect inflammatory changes. *Nocardia* infection of the spinal cord is rare and is typical in this case. Subcutaneous, soft tissue is a common invasive site, and abscess is the main manifestation [20]. Pathological tissue can be easily obtained through ultrasound and puncture. Pituitary infection usually presented as abscess formation, which is relatively rare, especially caused by *Nocardia* [21].

At present, the preferred method for *Nocardia* detection is 16S rRNA sequencing and other molecular techniques [22], such as metagenomic next-generation sequencing (NGS), which is a new technology that can be independently cultivated [8, 16]. The subcutaneous nodules in this case were confirmed to be *Nocardia* infection by direct microbial culture and mass spectrometry analysis, CSF culture was initially negative, followed by NGS testing positive for *Nocardia*. The changes of pulmonary lesions through short-term follow-up showed bacterial infection, which was consistent with the monism. The deficiency is that the pituitary gland has only plain MRI examination without enhanced, in combination with the systemic lesions and from the perspective of monism, considering the high possibility of infection of *Nocardia*, further follow-up and review will be conducted.

Sulfa drugs are the main drugs for the treatment of *Nocardia* [23]. But due to rising sulfonamide resistance and the diversity of bacteria that infect different individuals, some scholars now propose multi-drug combination therapy [24], such as Trimethoprim/sulfamethoxazole (TMP/SMZ). The treatment time depends on the patient’s basic physical condition and repair differences. If the infection is widespread and involves the central nervous system, treatment can take for at least a year [25]. After one month of the above treatment, the chest and back pain of the patient was significantly relieved. Sensory disturbance of the body was slightly relieved after therapy. There was no change in motor level of the lower extremities. Physical examination revealed symmetrically completely paralysis in the lower extremities, increased muscle tension, presence of knee and ankle reflexes, and the presence of bilateral extensor plantar reflex. The level of sensory disturbance of the body on the left side decreased to T7 level from T4 level at the time of admission.

Multisystem *Nocardia farcinica* infection is relatively rare. *Nocardia* is usually inhaled through the respiratory tract to pulmonary, and then to multiple organs through the blood, causing inflammatory changes or abscess formation. Until now, no case has been reported involving lung, spinal cord, skin and pituitary gland at the same time. *Nocardia* infection of spinal cord as the main manifestation is rare and requires sufficient attention. The diagnosis of *Nocardiosis* is difficult, and the prognosis of patients with spinal cord involvement is worse, so early diagnosis is particularly important. Through a comprehensive imaging examination, it is easy to find inflammatory signs, which will prompt the clinic.

## Data Availability

Data sharing is not applicable to this article as no datasets were generated or analyzed during the current study.

## Abbreviations

CSF: Cerebrospinal fluid
WBC: White blood cell
HIV: Human immunodeficiency virus
MRI: Magnetic resonance imaging
CT: Computed tomography
AIDS: Acquired immunodeficiency syndrome
NGS: Next-generation sequencing
TMP/SMZ: Trimethoprim/Sulfamethoxazole
